# Anionic effects on the structure and dynamics of water in superconcentrated aqueous electrolytes

**DOI:** 10.1039/c8ra09589b

**Published:** 2019-01-02

**Authors:** Sungho Han

**Affiliations:** CAE Group, Platform Technology Lab, Samsung Advanced Institute of Technology Suwon Gyeonggi 16678 Korea hellosungho@gmail.com

## Abstract

Dissolved ions in aqueous solutions are ubiquitous in a variety of systems and the addition of ions to water gives rise to dramatic effects on the properties of water. Due to a significant role of ions in the structure and dynamics of water, the ionic conditions, such as the ion type and concentration, have been considered as critical factors. Here we study the effects of anions on the structure and dynamics of water in aqueous electrolytes for various lithium salt concentrations *via* extensive molecular dynamics simulations. Our results demonstrate that a certain amount of salt is needed to show the different properties of water caused by the presence of different types of anion. Below the cutoff concentration, most features of water show the same characteristics in spite of the presence of different anions. In the superconcentrated limit, we find that full disruption of the hydrogen bond network between water molecules occurs for most anions investigated, indicating that the effect of the water–water interaction becomes negligible. However, a certain type of anion could enhance an ion-pairing of cations and anions and the water–water interaction remains considerable even in the superconcentrated limit. We further investigate the cationic and anionic hydration shell structures and dynamics, revealing their dependence on the anion type and the salt concentration. Finally, we observe that the anionic effects on water extend to the dynamics of water molecules, such as an anionic dependence of the onset of subdiffusive translation and anisotropic rotation.

## Abstract 2

The effects of anions on the properties of water are examined for various salt concentrations.

## Graphical abstract

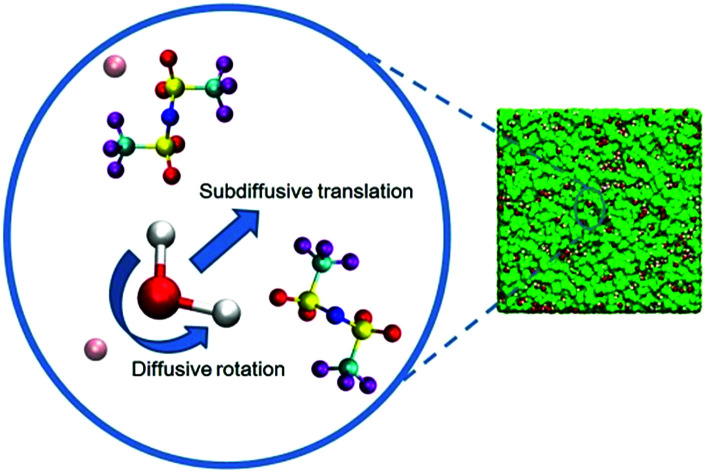

## 1 Introduction

The ability to form hydrogen-bonds (HBs) makes water unique among other liquids – with properties such as unusual temperature dependence of density and thermodynamic response functions, high boiling and melting temperatures and high surface tension.^1^ In recent years, furthermore, it has been proposed by computer simulations that water in the deeply supercooled regime could exhibit two distinct liquid properties of low-density water and high-density water, and they are separated by a first-order transition line terminating at the hypothesized second critical point.^2^ However, confirming the liquid–liquid phase transition scenario has been an experimentally challenging task due to the restriction of experimental approaches, also known as “no-man’s land”, since the nucleation of water occurs on a much shorter timescale than experimentally accessible timescales. As a result, the liquid–liquid phase transition scenario of water still remains controversial.^3–5^ Instead of direct exploration, existence of the Widom line, the locus of maximum thermodynamic response functions beyond a critical point, has been intensively explored in experiments and simulations.^6–10^ The remarkable and unusual properties of water are believed to originate from its complex HB network related to the tetrahedral structure, emphasizing the role of the HB network in the properties of water.

Water is ubiquitous in nature and it mostly exists in complex forms with ions rather than as neat water. Complex forms of water with ions commonly exist in many chemical and biological systems.^11,12^ The influence of ions on the structure and dynamics of water is of great importance for understanding their roles in chemical and biological processes such as protein stability, cell membrane transport, and aerosol formation.^13^ Hence the effects of ions on the structure and dynamics of water have been extensively investigated both experimentally and theoretically.^14–24^ For example, there is a subject of ongoing discussion about the spatial extent of the influence of the dissolved ions on the structure of the surrounding water molecules in solutions. Recent studies have shown that long-range orientational order between water molecules exists in dilute salt solutions.^25,26^ The ionic effects on water are often described in terms of a concept of the structure-maker and structure-breaker,^27^ where ions could participate in either enhancing or weakening the HB network of water. A celebrated example is the Hofmeister series,^28^ a classification of ions in order of their ability to salt out or salt in proteins. Although the concept of the structure-maker/breaker of ions has been widely accepted, the effects of ions on the structure of water remain elusive. Some results are in good agreement with the concept, but some are not.^17,18^ Furthermore, many studies have shown that the ionic effects on the properties of water strongly depend on the type of ions. Experimental and simulation studies have shown that the dynamics of water can be suppressed or enhanced by the presence of a different type of ions, which is induced by the subtle change in the structure of water by the ions.^29,30^

Superconcentrated conditions in electrolytes have not gained much attention due to the lack of their practical applications. Recently, however, a series of studies has proposed that superconcentrated aqueous electrolytes could simultaneously enhance both the performance and safety of lithium ion batteries.^31–35^ The usage of aqueous electrolytes in lithium ion batteries has been limited by the narrow electrochemical stability of water.^36^ A water molecule is decomposed at around 1.23 volts, which is far below the practical demand for battery operations. Recent studies have shown that lithium ion batteries of 2.3 volts can be stably operated with superconcentrated aqueous electrolytes up to 1000 cycles with nearly 100% coulombic efficiency.^32–34^ They have also shown that an extremely high concentration of a lithium bis(trifluoromethanesulfonyl)imide salt (called ‘water-in-salt’) could help the batteries to be operated at up to 3 volts. In superconcentrated aqueous electrolytes, the interaction between salt and water would enormously increase and the properties of water would predominantly depend on the nature of interactions with cations and anions. For the electrolytes of lithium ion batteries, various anions in the lithium salt have been introduced and tested so far.^37–39^ However, the effects of anions on the structure and dynamics of water are far from being fully understood.^15^

In this work, we explore how the existence of different anions will affect the structural and dynamic properties of water in aqueous electrolytes for various salt concentrations, focusing on superconcentrated conditions. For the sake of it, we consider four different anions, as a counterion of a lithium ion, – bis(trifluoromethanesulfonyl)imide (TFSI^−^), bis(fluorosulfonyl)imide (FSI^−^), trifluoromethanesulfonate (OTf^−^ or triflate) and nitrate (NO_3_^−^), according to their sizes. For each anion, we construct the systems with six salt concentrations: 1 M, 2 M, 5 M, 10 M, 15 M and 20 M. Especially, we focus on investigating the HB network structure and HB dynamics of water in the presence of different anions. In addition, we investigate the hydration shell structure and dynamics of an Li^+^ ion and four different anions. Finally, we examine the dependence of the translational and rotational dynamics of water molecules on the type of anion.

## 2 Methods

We performed molecular dynamics (MD) simulations of aqueous electrolyte solutions consisting of a lithium salt in water modeled with the extended simple point charge (SPC/E) model.^40^ For comparison of anionic effects, we modeled four different anions: TFSI^−^, FSI^−^, OTf^−^ and NO_3_^−^. We investigated the systems with six different salt concentrations: 1, 2, 5, 10, 15 and 20 M. The number of water molecules was *N*_W_ = 5832 and the number of the salt was *N*_S_ = 105 up to 2100, depending on the salt concentration. We carried out all simulations using the MD simulation package, LAMMPS.^41^ We implemented the OPLS/AA force field to describe the molecular interaction of the anions.^42^ We used the combination rule of Lorentz–Berthelot for the intermolecular interactions. We computed the long-range interactions using the particle–particle particle–mesh (PPPM) algorithm. The simulations were performed initially in the *NPT* ensemble and then in the *NVT* ensemble, where *N*, *V*, *P* and *T* are the number of molecules (*N*_W_ + *N*_S_), the volume, the pressure and the temperature, respectively. We kept the temperature and pressure constant *via* the Nóse–Hoover thermostat and barostat during the simulations. We applied periodic boundary conditions in all three directions of the simulation box. We used 1 fs as the timestep of the simulation. For each salt concentration, we ran MD simulations of 50 ns for the equilibration and 30 ns for the data collection. Initially, we prepared the random configuration and then increased the temperature up to 400 K to mix the system properly. After then, we decreased the temperature to the target temperature, 300 K, and finally equilibrated the system.

## 3 Results and discussion

### 3.1 Structure and hydrogen-bond networks of water

**Fig. 1:**
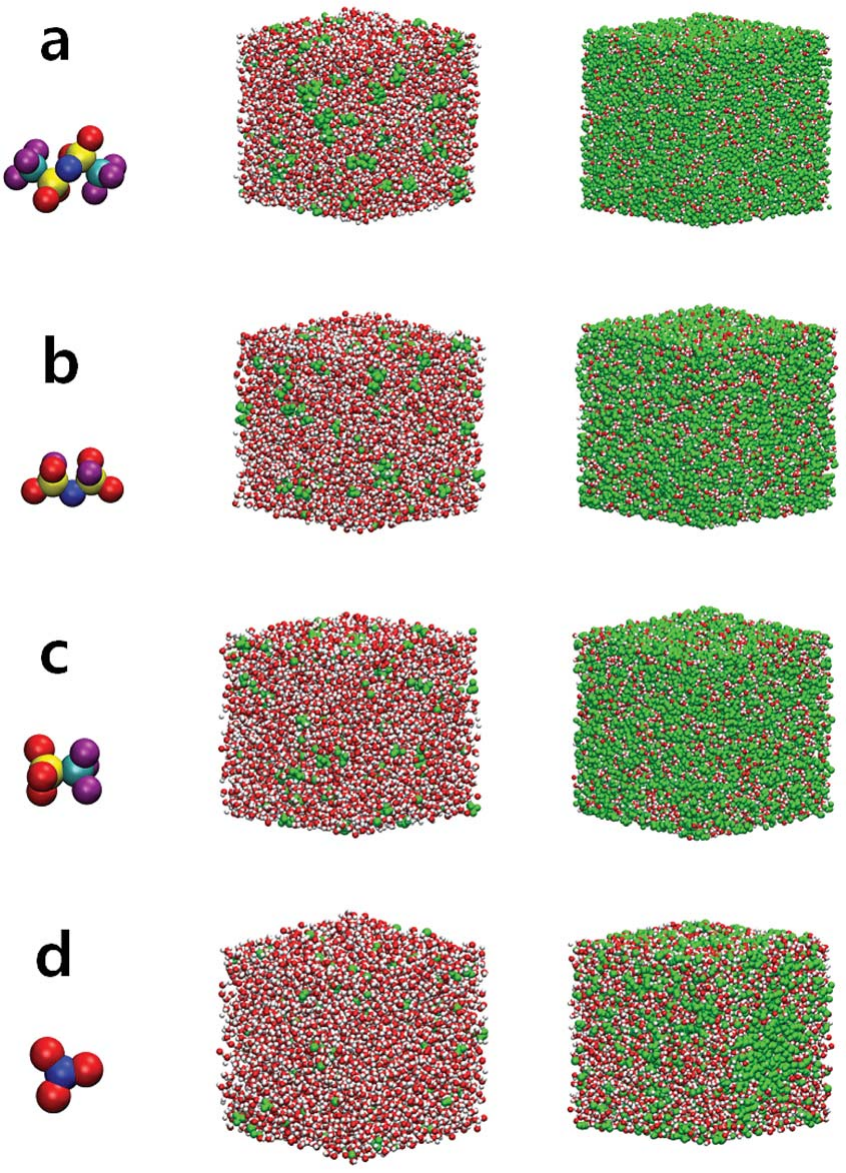
Configurations of electrolytes at low and high salt concentrations for different anions. Shown are snapshots of aqueous electrolytes at the salt concentrations of 1 M (left) and 20 M (right) for (a) TFSI^−^, (b) FSI^−^, (c) OTf^−^ and (d) NO_3_^−^. The green atoms in the snapshots represent cations and anions. For the molecular description of each anion, the red, yellow, blue, cyan and purple atoms represent O, S, N, C and F atoms, respectively.

At low and extremely high salt concentrations, the surrounding conditions of water molecules are quite different and a dominant factor for the structure and dynamics of water would be either the water–water interaction or the ion–water interaction in both concentration limits. In Fig. 1, we present the configurations of four different aqueous electrolytes at low and extremely high salt concentrations. The configurations for all anions at 1 M show that most water molecules are surrounded by other water molecules and a relatively small fraction of them directly interact with cations and anions. In the superconcentrated limit, on the contrary, almost all water molecules are surrounded by ions and directly interact with them. Thus, the interactions of water with cations and anions would be key to determining the properties of water in the superconcentrated limit. It would be of great importance to understand how the properties of water depend on the type and amount of anions. Note that for NO_3_^−^ the configuration at 20 M is different from the configurations of the other anions. This shows the enhanced ion-pairing of Li^+^ ions and NO_3_^−^ ions, indicating the low solubility of the lithium salt in water.

**Fig. 2:**
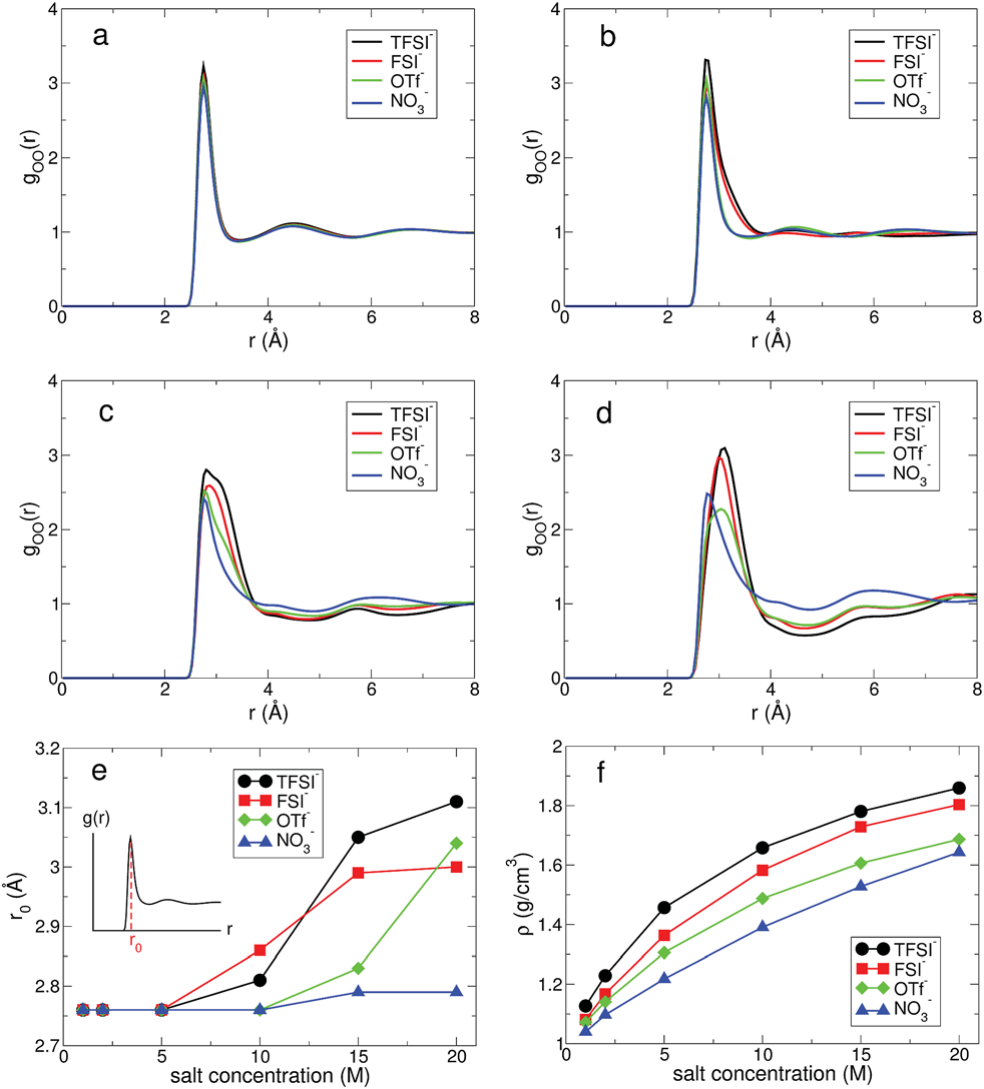
The structure of water in aqueous electrolyte solutions. The oxygen–oxygen radial distribution function (RDF) *g*_OO_(*r*) between water molecules for different anions at the salt concentrations of (a) 1 M, (b) 5 M, (c) 10 M and (d) 20 M. (e) The position *r*_0_ of the first peak in *g*_OO_(*r*) as a function of salt concentration for different anions. (f) The total density *ρ* of aqueous solutions as a function of salt concentration for various anions.

First, we investigated the structure of water by calculating the oxygen–oxygen radial distribution function (RDF) *g*_OO_(*r*) between water molecules.^24,43,44^ In Fig. 2, we present *g*_OO_(*r*) for four different anions. The RDFs at 1 M show no difference in the presence of the different anions. Namely, all RDFs fall onto the same curve. This tells us that the water–water interaction at 1 M remains dominant for the structure of water. As the salt concentration increases, we observe growing differences in *g*_OO_(*r*) with respect to the anion types. When the salt concentration becomes 5 M, the first peak of *g*_OO_(*r*) for TFSI^−^ and FSI^−^ broadens, even though the peak position does not change from *g*_OO_(*r*) at 1 M. At the extremely high concentration of 20 M, *g*_OO_(*r*) of the four different anions become clearly distinguishable from each other. The position *r*_0_ of the first peak in *g*_OO_(*r*) for TFSI^−^ and FSI^−^ shows the increase when the salt concentration becomes 10 M, whereas *r*_0_ for OTf^−^ and NO_3_^−^ starts to increase above 15 M. Thus, we conclude that the cutoff concentration generating the structural change in water depends on the type of anion. Note that the *r*_0_ for NO_3_^−^ increases by about 0.03 Å at 20 M and it is relatively small compared to the other anions. The structure of water in the environment with NO_3_^−^ is not affected much by increasing the salt concentration.

**Fig. 3:**
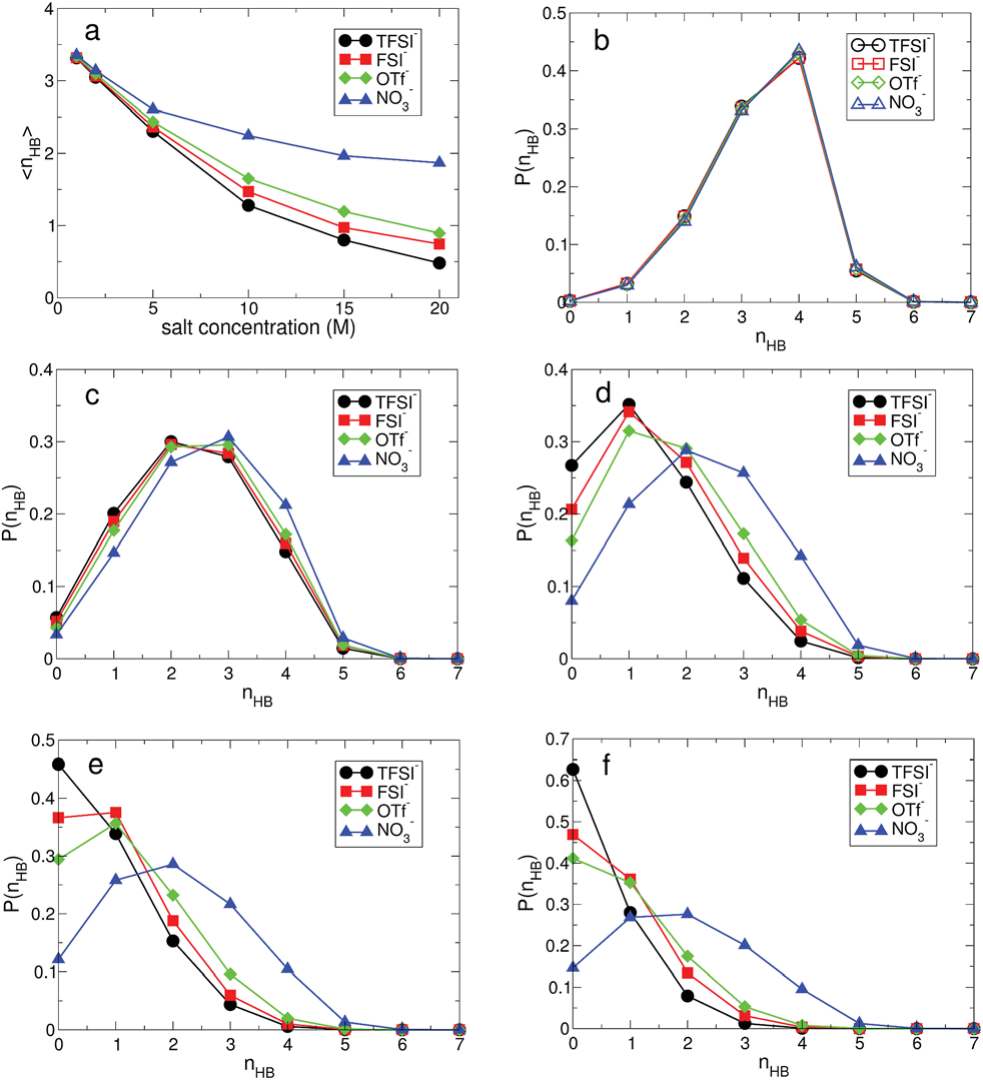
The structure of the hydrogen-bond network between water molecules. (a) Average hydrogen-bond number 〈*n*_HB_〉 per molecule between water molecules as a function of salt concentration for different anions. The probability function *P*(*n*_HB_) of a water molecule having *n*_HB_ hydrogen bonds at the salt concentrations of (b) 1 M, (c) 5 M, (d) 10 M, (e) 15 M and (f) 20 M for each anion.

The structural change in *g*_OO_(*r*) we observed continues to the complex HB network in water. To explore changes in the HB characteristics of water, we adopted the traditional geometric definition of HB.^24,45–47^ This describes that the two tagged water molecules are considered to be hydrogen-bonded if simultaneously the distance between two oxygen atoms is less than 3.5 Å, and the angle between intra O–H and O⋯O is less than 30°. In Fig. 3, we present the average HB number and its distribution. Note that the HB in water denotes only the HB between water molecules. At 1 M, the average HB number 〈*n*_HB_〉 per molecule between water molecules is approximately 〈*n*_HB_〉 = 3.32–3.35, showing a small reduction in the HB number compared to bulk water, 〈*n*^bulk^_HB_〉 = 3.40–3.60.^24,45–47^ 〈*n*_HB_〉 gradually decreases as the salt concentration increases. When it becomes 20 M, 〈*n*_HB_〉 for all anions except NO_3_^−^ is less than 1.0, which means that one molecule acts as either an HB donor or an HB accepter. Otherwise, the molecules do not participate in forming the HB network of water at all. This indicates that connectivity of the HB network between water molecules is fully disrupted by the existence of ions in the superconcentrated limit. In contrast, 〈*n*_HB_〉 for NO_3_^−^ at 20 M is about 2.0, indicating that a water molecule still plays the roles of both an HB donor and an HB acceptor, simultaneously. This different behavior in the presence of NO_3_^−^ stems from the low solubility of the lithium salt in water.

For detailed information on the HB number of water, we calculated the probability function *P*(*n*_HB_) for a water molecule to have *n*_HB_ hydrogen-bonding numbers.^24^ In Fig. 3, we present *P*(*n*_HB_) for various salt concentrations. At 1 M, *P*(*n*_HB_)s for all anions overlap with each other, which is the same behavior as found in *g*_OO_(*r*). This means that the HB network in water for low concentrations does not depend on the type of anion. Upon increasing the salt concentration, *P*(*n*_HB_) shows dependence on the anion species. For TFSI^−^, at 20 M, more than 60% of water molecules do not form HBs with the other water molecules at all. For FSI^−^ and OTf^−^, about half of the water molecules at 20 M do not participate in forming the HB network in water at all. For NO_3_^−^, we find different behavior in *P*(*n*_HB_) above 10 M, compared to the other anions. Even at the salt concentration of 20 M, *P*(*n*_HB_) shows a maximum at *n*_HB_ = 2.0, indicating the wide span of the HB network in water. The disruption of the HB network in water is the smallest in the presence of NO_3_^−^. In the superconcentrated limit, hence, the anion type could affect the formation of the HB network in water. Previously, an experimental study has shown that ions give negligible effects on the HB structure of water.^48^ However, the concentration of ions in this study was not within the range of the superconcentrated conditions.

### 3.2 Hydrogen-bond dynamics of water

Next, we examine the fast and slow HB dynamics of water molecules to understand how the breaking and forming of HB between water molecules occur on different timescales for various anionic environments.^49^ First of all, we describe the fast HB dynamics of water *via* the HB residence time distribution^24,45,47^ defined as

**Fig. 4:**
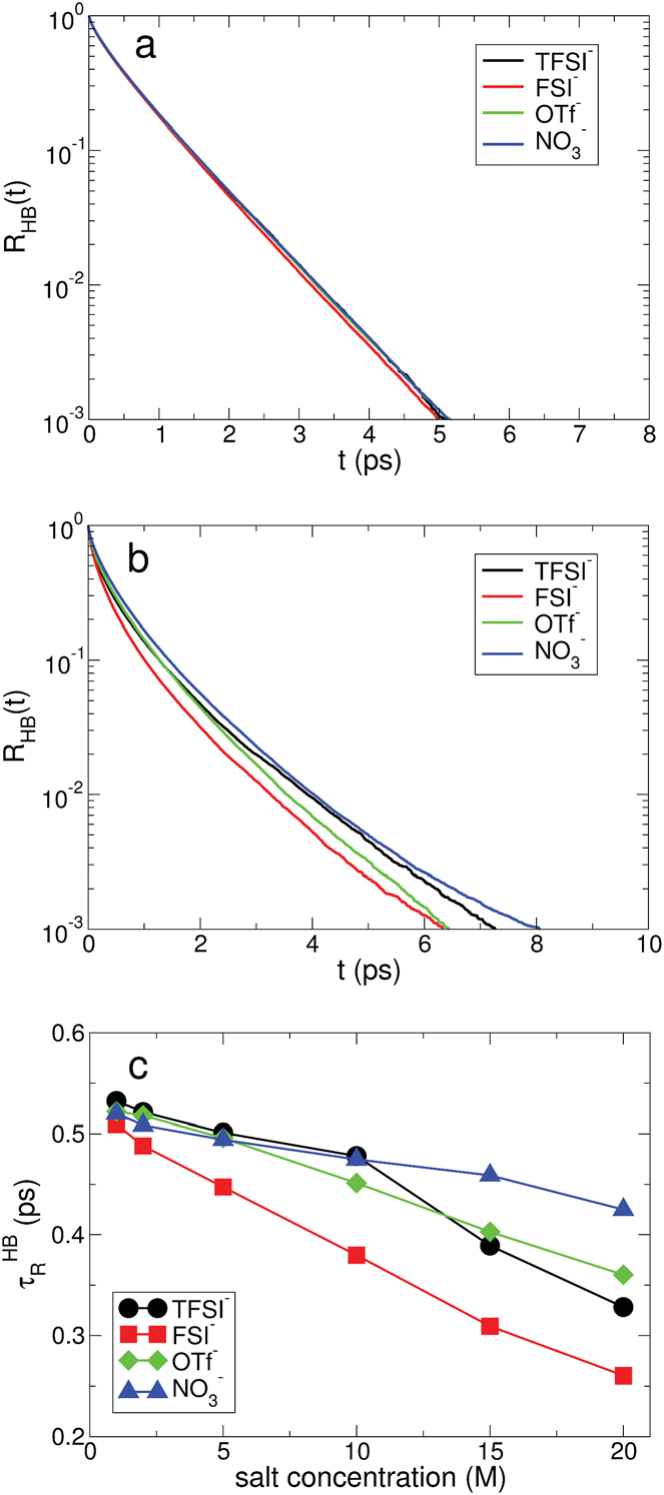
The hydrogen-bond dynamics on a short timescale. The hydrogen-bond residence time distribution *R*_HB_(*t*) of water molecules at the salt concentrations of (a) 1 M and (b) 20 M. (c) The characteristic HB residence time *τ*^HB^_R_ as a function of salt concentration for different anions.

1 *R*_HB_(*t*) ≡ 〈*Θ*(*t*^HB^_b_ − *t*)〉,

where *Θ*(*t*) is the Heaviside step function, *t*^HB^_b_ is the first-passage time for an HB to be broken, and 〈⋯〉 represents an ensemble average. In this definition, *R*_HB_(*t*) considers only intact HBs for the given time interval, which is often interpreted as the continuous distribution of the HB lifetime.^24,45,47^ The fast HB dynamics of water is known to be associated with the librational motion (the hidden rotation).^45,46^ In Fig. 4, we present *R*_HB_(*t*) at low and high salt concentrations. At the low concentration of 1 M, *R*_HB_(*t*) does not show any differences for the different anionic environments. On the contrary, *R*_HB_(*t*) at 20 M exhibits different temporal behaviors in the fast kinetics of HB due to the presence of the different types of anion. To quantify the time evolution of *R*_HB_(*t*) in terms of a single variable, it is appropriate to introduce the characteristic residence time *τ*^HB^_R_ of HB, defined as the time required for *R*_HB_(*t*) to decay by a factor of *e*.^24,47^ In Fig. 4(c), *τ*^HB^_R_ for all anions decreases as the salt concentration increases, indicating the faster librational motion of water molecules with the larger salt concentration. The magnitude of *τ*^HB^_R_ at 1 M is TFSI^−^ > OTf^−^ > NO_3_^−^ > FSI^−^. At 20 M, the magnitude of *τ*^HB^_R_ is different: NO_3_^−^ > OTf^−^ > TFSI^−^ > FSI^−^. For all salt concentrations investigated, the fast HB dynamics of water molecules in the environment with FSI^−^ occurs at the shortest time. This suggests that the rotational dynamics of water molecules related with the librational motion would be the fastest in the anionic environment with FSI^−^, as we will see later, so that the breaking and forming of HB in water in association with the thermal fluctuations occur the most frequently with FSI^−^ among the four anions. For NO_3_^−^, the difference in *τ*^HB^_R_ at the salt concentrations of 1 M and 20 M is the smallest, indicating that the dependence of the fast HB dynamics on the salt concentration is weak.

**Fig. 5:**
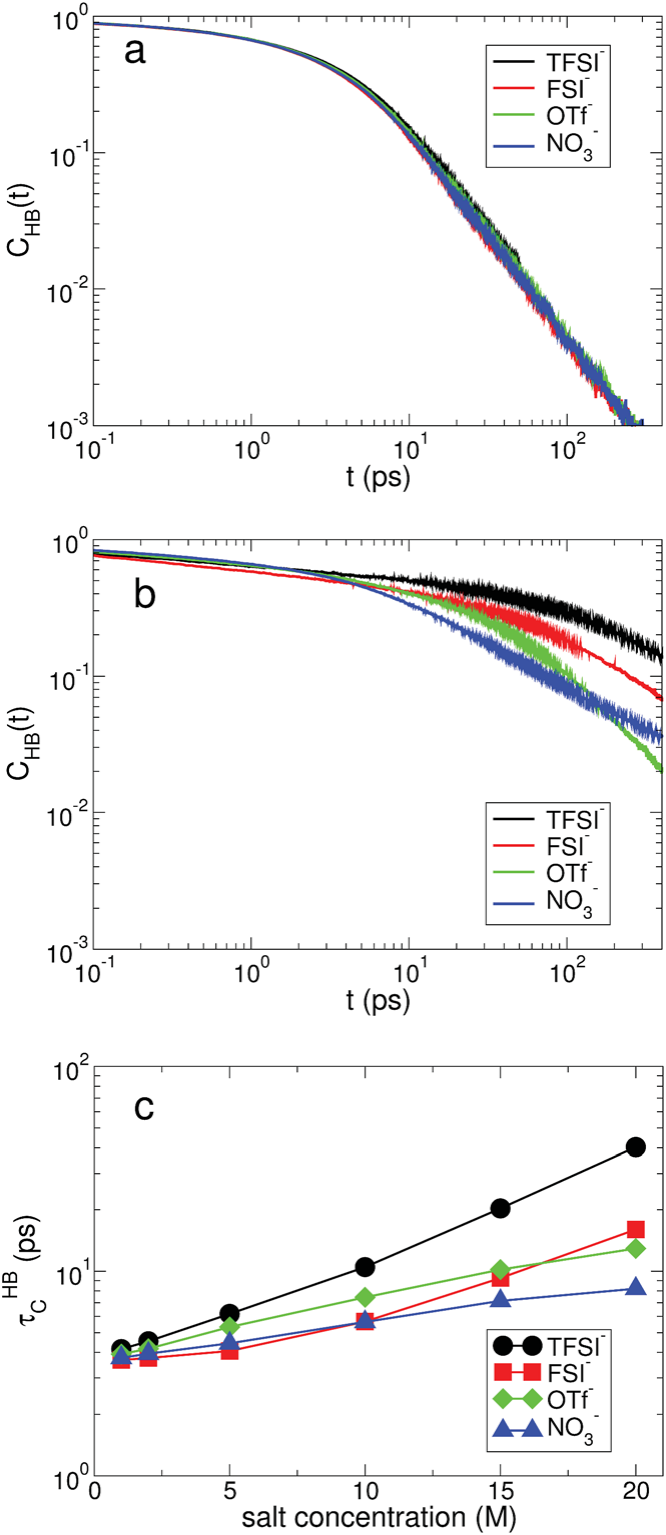
The hydrogen-bond dynamics on a long timescale. The hydrogen-bond correlation time distribution *C*_HB_(*t*) of water molecules at the salt concentrations of (a) 1 M and (b) 20 M. (c) The characteristic HB correlation time *τ*^HB^_C_ as a function of salt concentration for different anions.

For the slow kinetics of HB, we define the HB correlation time distribution *C*_HB_(*t*)^24,45–47,50^ as2
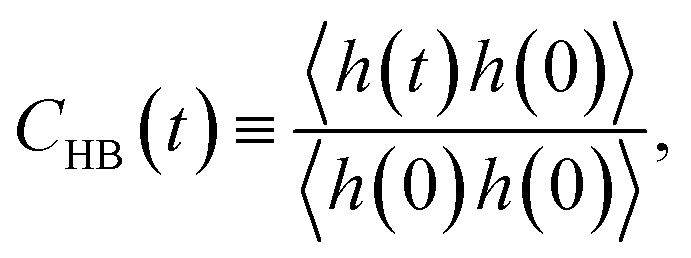
where *h*(*t*) is unity when the two tagged water molecules are hydrogen-bonded at time *t*, and *h*(*t*) is zero otherwise. *C*_HB_(*t*) indicates the conditional probability that an HB remains intact at time *t*, given that it was intact at time *t* = 0. In contrast to *R*_HB_(*t*), *C*_HB_(*t*) does not consider any breaking of HBs at intermittent times between time zero and *t*, thus showing the discontinuous distribution of the HB lifetime.^47^ The breaking of HB represented by *C*_HB_(*t*) is closely connected with the diffusion of water molecules instead of the thermal fluctuations. In Fig. 5, we observe that *C*_HB_(*t*) at 1 M does not show anionic dependence of the HB correlation time. In the superconcentrated limit, however, *C*_HB_(*t*) clearly shows different temporal behaviors in the presence of the different types of anion. In Fig. 5(c), we present the characteristic HB correlation time *τ*^HB^_C_ defined as the time needed for *C*_HB_(*t*) to decay by a factor of *e*.^24,45,47^ For all anions, *τ*^HB^_C_ exhibits an exponential increase upon increasing the salt concentration. Since the slow HB dynamics of water is closely related to the diffusion of water, the exponential increase in *τ*^HB^_C_ effectuates an exponential increase in the translational diffusion of water upon increasing the salt concentration, as we will show later. The effects of anions on the slow HB dynamics dramatically grow in the superconcentrated limit. The increasing rates of *τ*^HB^_C_ with respect to the salt concentration for TFSI^−^ and FSI^−^ are much larger than for OTf^−^ and NO_3_^−^, suggesting a possible relation of the HB dynamics of water with anion size.

The effects of the salt concentration on the forming and breaking of HB in water result in the two different behaviors in the fast and slow HB dynamics for all anions. Whereas *τ*^HB^_R_ gradually decreases upon increasing the salt concentration, *τ*^HB^_C_ increases exponentially. This tells us that two different timescales would be needed to fully characterize the HB dynamics of water in aqueous electrolyte solutions, on which the dependences of the HB dynamics on the salt concentration show opposite behaviors. Since the structure and dynamics of HB have deep impacts on the dynamical properties of water molecules, one can make a connection of the above results with the dynamical behaviors of water with respect to the salt concentration: the faster thermal fluctuations (possibly related to the rotational motion) and the much slower translational motion with the increasing salt concentration. Hence one can understand the dynamics of water with a molecular picture that a water molecule rotates faster on a certain molecular axis but it translates much slower with the given surrounding conditions for higher salt concentrations. We will see this later in detail. For NO_3_^−^, the changes in both *τ*^HB^_R_ and *τ*^HB^_C_ according to the salt concentration are quite small compared with the other anions. The result of 〈*n*_HB_〉 ∼ 2 for NO_3_^−^ confirms that the water–water interaction is still influential on the properties of water even in the superconcentrated limit, so that the effect of NO_3_^−^ on the HB dynamics of water is relatively weaker than the other anions due to the enhanced ion-pairing.

### 3.3 Cationic and anionic hydration shells – structure and dynamics

**Fig. 6:**
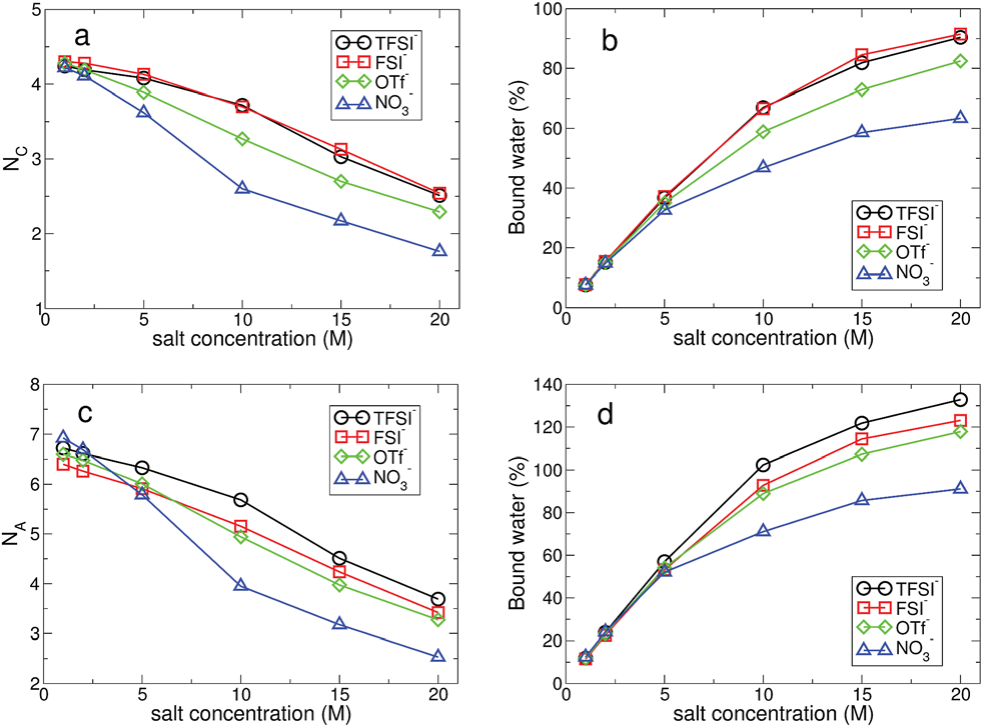
Cationic and anionic hydration shell structures. (a) The average number *N*_C_ of water molecules forming a cationic hydration shell per cation as a function of salt concentration for different anions. (b) The ratio of water molecules bound within the cationic hydration shell as a function of salt concentration for different anions. (c) The average number *N*_A_ of water molecules forming an anionic hydration shell per anion as a function of salt concentration for different anions. (d) The ratio of water molecules bound within the anionic hydration shell as a function of salt concentration for different anions.

Since the ion–water interactions are of great importance to the properties of water in the superconcentrated limit, it is natural to investigate how water molecules interact with ions by considering the structure and dynamics of hydration regarding the cationic and anionic hydration shells. For the structural properties of the cationic hydration shell, we first calculated the lithium solvation number *N*_C_ of water, which is defined as the number at the first plateau in the cumulative coordination number,^24,51^3
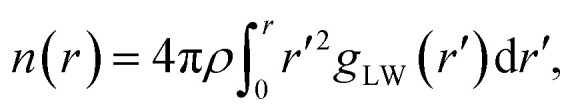
where *g*_LW_(*r*) is the RDF between Li^+^ ions and water molecules and *ρ* is the density. For the anionic hydration shell structure, we consider the average number *N*_A_ of water molecules hydrogen-bonded with the oxygen atoms of each anion. To describe the HB between the anions and water, we use the same geometric definition of HB as in water molecules. In Fig. 6, we present *N*_C_ and *N*_A_ as a function of salt concentration for all anions. At 1 M, we find a small difference in *N*_A_, whereas *N*_C_ is almost the same for all anions. On average, a Li^+^ ion is surrounded by approximately 4.3 water molecules independent of the type of anion. For an anion, it is hydrogen-bonded with 6.4 ∼ 7.0 water molecules on average, depending on the type of anion. The difference in *N*_A_ comes from the different number of oxygen atoms for each anion. As the salt concentration increases, both *N*_C_ and *N*_A_ gradually decrease, indicating that the ion-pairing of cations and anions increasingly occurs by expelling water molecules from their hydration shells. When the salt concentration becomes 20 M, both *N*_C_ and *N*_A_ for NO_3_^−^ decrease by the largest amount. For TFSI^−^ and FSI^−^, interestingly, *N*_C_ shows the same number at the same salt concentration. Namely, the structure of the cationic hydration shell remains the same by switching TFSI^−^ to FSI^−^ and *vice versa*.

Now we can classify water molecules into those bound within the hydration shell of ions (bound) and those outside of it (free). In Fig. 6, we present the fraction of bound water molecules for each hydration shell for all anions. Although the average number of water molecules per ion within each hydration shell seems to decrease upon increasing the salt concentration, the total number of bound water molecules increases due to the increase in the number of ions. At 1 M, the fraction of bound water molecules in the cationic hydration shell is below 10% for all anions, and then at 20 M it exceeds 90% for TFSI^−^ and FSI^−^. For NO_3_^−^, the fraction of bound water molecules is around 60% and it is the lowest among the anions in the superconcentrated limit. For the anionic hydration shell, the fraction of bound water molecules at 20 M exceeds 100% for the three anions of TFSI^−^, FSI^−^ and OTf^−^, indicating that one water molecule is hydrogen-bonded with more than one anion. For NO_3_^−^, however, it is below 100%. This confirms that the water–water interaction in the anionic environment of NO_3_^−^ remains significant and lithium nitrate has a relatively low solubility limit in water among the four anions.

The water molecules hydrating ions have a finite lifetime in their residence within the hydration shell. The key idea to describe the exchange dynamics should be how long water molecules reside within the hydration shell of each ion.^14,52,53^ The residence time of water molecules within the hydration shell, also known to represent the rigidity of the hydration shell, plays an important role in the transport of ions.^14,51^ We examine the fast and slow kinetics of the exchange dynamics of water molecules in the hydration shell, *R*(*t*) and *C*(*t*), which are defined in the same ways as in eqn (1) and (2), respectively. The mechanisms of the two exchange dynamics are different. The fast exchange dynamics of water is associated with the thermal fluctuations in the motion of water molecules and the slow exchange dynamics is closely connected with the diffusive motions of both ions and water. For a Li^+^ ion, we describe the fast and slow exchange dynamics in terms of the cationic residence time and correlation time distributions of water molecules, *R*_LW_(*t*) and *C*_LW_(*t*), respectively. We consider that a bond between a Li^+^ ion and the oxygen atom of a water molecule is broken if the distance between them exceeds 2.7 Å, the approximate size of the first hydration shell. Similarly, for the anionic hydration dynamics, we calculate the fast and slow HB dynamics of water molecules with the oxygen atoms of anions, *R*_AW_(*t*) and *C*_AW_(*t*), respectively.

**Fig. 7:**
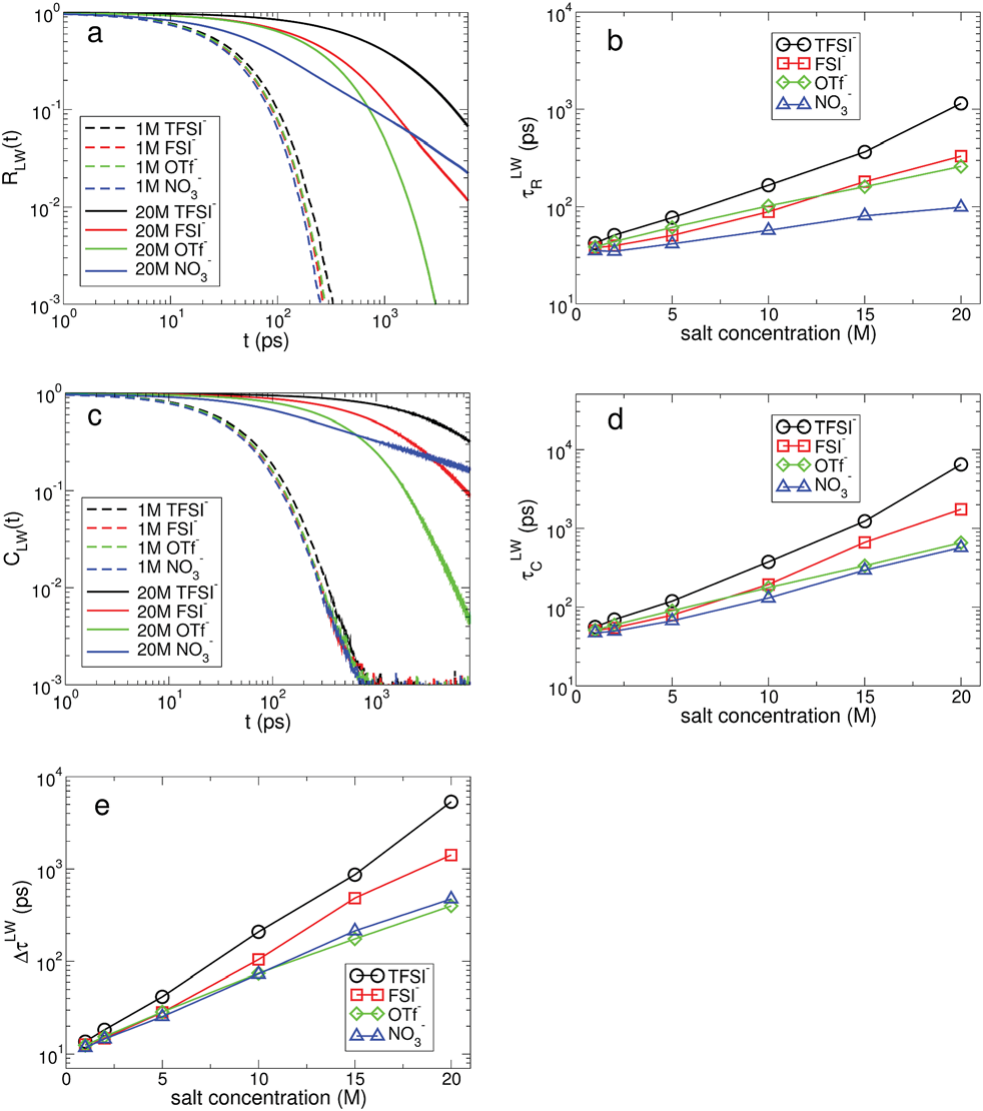
Cationic hydration dynamics. (a) The residence time distribution *R*_LW_(*t*) of water molecules within the cationic hydration shell for different anion types and salt concentrations. (b) The characteristic residence time *τ*^LW^_R_ as a function of salt concentration for different anions. (c) The residence correlation time distribution *C*_LW_(*t*) of water molecules within the cationic hydration shell for different anion types and salt concentrations. (d) The characteristic residence correlation time *τ*^LW^_C_ as a function of salt concentration for different anions. (e) The difference Δ*τ*^LW^ in two different characteristic residence times of short and long timescales, defined as Δ*τ*^LW^ ≡ *τ*^LW^_C_ − *τ*^LW^_R_.

In Fig. 7, we present the *R*_LW_(*t*) and *C*_LW_(*t*) at low and high salt concentrations for the different types of anion. At low concentration, we find a small difference in both distributions for all anions. In other words, the effect of anions on the cationic hydration dynamics of water is weak at low concentration and the exchange dynamics of water in the cationic hydration shell is mostly affected by the other water molecules staying near the first hydration shell. Both *R*_LW_(*t*) and *C*_LW_(*t*) exhibit slower decaying behaviors with higher salt concentrations. To characterize the temporal behaviors of *R*_LW_(*t*) and *C*_LW_(*t*) in terms of a single variable, we introduce the characteristic residence and correlation times, *τ*^LW^_R_ and *τ*^LW^_C_, defined as the times required for *R*_LW_(*t*) and *C*_LW_(*t*) to decay by a factor of *e*, respectively. Both the characteristic times of *τ*^LW^_R_ and *τ*^LW^_C_ increase upon increasing the salt concentration for all anions. For cationic hydration dynamics, in contrast to the HB dynamics of water, the fast and slow hydration dynamics show the same characteristics with respect to the salt concentration. Interestingly, the difference in the two characteristic times, Δ*τ*^LW^ ≡ *τ*^LW^_C_ − *τ*^LW^_R_, shows an exponential change with a change in the salt concentration. As a result, this suggests that at low concentration the fast and slow cationic hydration dynamics would occur almost concurrently, but for the increasing salt concentration the timescales needed for describing the two hydration dynamics become separate and the two dynamics occur with a certain time gap.

**Fig. 8:**
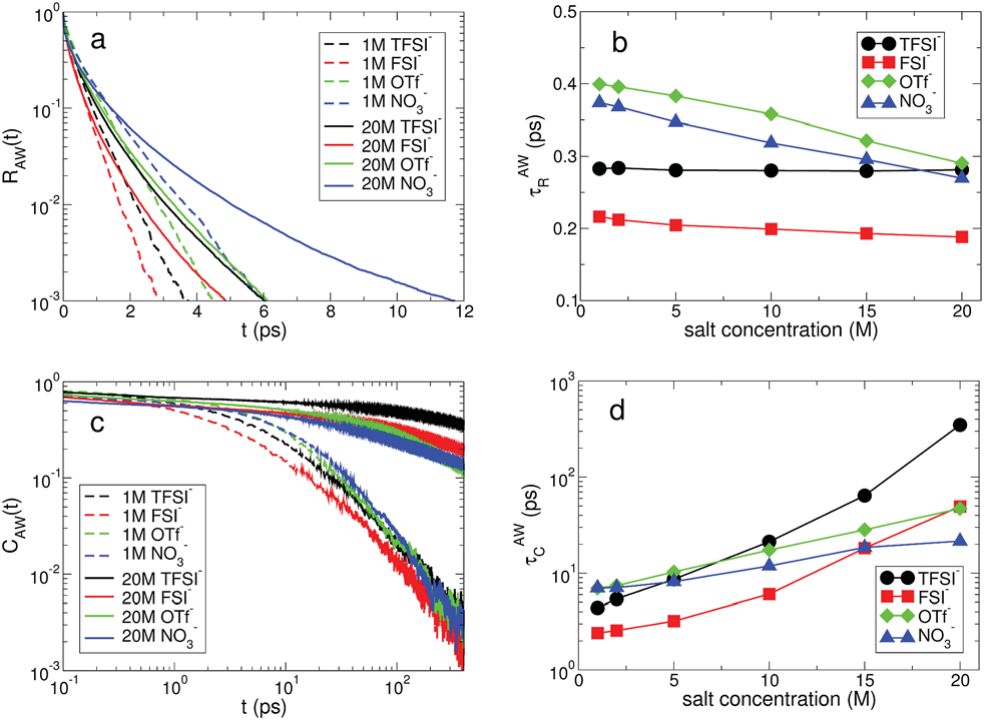
Anionic hydration dynamics. (a) The hydrogen-bond lifetime distribution *R*_AW_(*t*) of water molecules hydrogen-bonded with anions at salt concentrations of 1 M and 20 M. (b) The characteristic HB residence time *τ*^AW^_R_ as a function of salt concentration for different anions. (c) The hydrogen-bond correlation time distribution *C*_AW_(*t*) of water molecules hydrogen-bonded with anions at salt concentrations of 1 M and 20 M. (d) The characteristic correlation time *τ*^AW^_C_ as a function of salt concentration for different anions.

For the anionic hydration dynamics, the behaviors of the fast and slow hydration dynamics are slightly different from those of the cationic hydration dynamics. In Fig. 8, we present the *R*_AW_(*t*) and *C*_AW_(*t*) at low and high salt concentrations for the different types of anion. Even at low concentration, we find that recognizable differences in *R*_AW_(*t*) and *C*_AW_(*t*) are observed due to the presence of the different types of anion. To characterize the temporal behaviors of *R*_AW_(*t*) and *C*_AW_(*t*), we also introduce the characteristic HB residence and correlation times, *τ*^AW^_R_ and *τ*^AW^_C_, defined in the same way as in *τ*^LW^_R_ and *τ*^LW^_C_. For the fast anionic hydration dynamics, we can categorize the behavior of *τ*^AW^_R_ into two groups: OTf^−^ and NO_3_^−^ belong to the first group and the second group contains TFSI^−^ and FSI^−^. We find that *τ*^AW^_R_ of the second group is almost unchanged with respect to a change in the salt concentration, whereas the first group shows a gradual change in *τ*^AW^_R_. This means that the fast HB kinetics with the TFSI^−^ and FSI^−^ anions is insensitive to the salt concentration for most water molecules, whereas the breaking and forming of HB with OTf^−^ and NO_3_^−^ occur at a faster time for the higher salt concentration. On the other hand, the slow HB kinetics with anions occurs in a different way. We can still categorize the behavior of *τ*^AW^_C_ into the same two groups, but their behavior is different from that for *τ*^AW^_R_. *τ*^AW^_C_ for TFSI^−^ and FSI^−^ abruptly increases upon increasing the salt concentration, whereas *τ*^AW^_C_ for OTf^−^ and NO_3_^−^ increases by relatively smaller values. By a direct connection of the slow anionic hydration dynamics with the diffusive motion of water molecules, our results reveal that the translational motion of water molecules abruptly slows down with TFSI^−^ and FSI^−^ upon increasing the salt concentration. As a result, the escape time of water molecules from the anionic hydration shell dramatically increases and the anionic hydration shell becomes more rigid on a long timescale. For the anionic hydration dynamics, we find that there are two timescales needed to describe the dependence of the hydration dynamics on the salt concentration, which are similar to the HB dynamics of water but in contrast to the cationic hydration dynamics.

### 3.4 Dynamical properties of water molecules

Lastly, we investigate how the existence of different anions would affect the translational and rotational dynamics of water molecules for increasing salt concentrations. First, we calculate the translational mean square displacement (TMSD), defined as^24,43,44,51,54,55^4
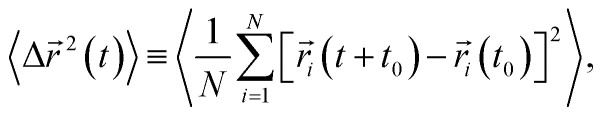
where 〈⋯〉 represents an ensemble average. For the rotational dynamics, we obtain the TMSD analogue of the rotational mean square displacement (RMSD) as follows.^24,56^ First, we quantify the rotational motion of a water molecule using the normalized polarization vector *H⃑*(*t*). Its direction is defined as the vector from an oxygen atom to the midpoint of the line joining two hydrogen atoms. For a given time interval δ*t*, the vector *H⃑*(*t*) will span the angle of δ*φ* ≡ cos^−1^[*H⃑*(*t* + δ*t*)·*H⃑*(*t*)]. Thus, we are able to define an angle vector δ*φ⃗*(*t*) at time *t*. The magnitude of the angle vector is |δ*φ⃗*(*t*)| ≡ δ*φ* and the direction of the angle vector is equal to *H⃑*(*t*) × *H⃑*(*t* + δ*t*). Finally, we obtain the angle vector *φ⃗*(*t*) by summing the angular velocity δ*ω⃗*(*t*) over time *t*,5
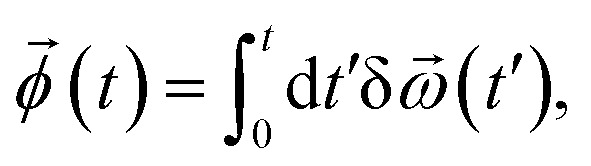
where the angular velocity is defined as δ*ω⃗*(*t*) ≡ δ*φ⃗*(*t*)/δ*t*. The form of the angle vector *φ⃗*(*t*) allows us to keep track of a trajectory of the angle vector *φ⃗*(*t*) as a function of time *t*, so that it is possible for us to calculate the RMSD, defined as^24,56,57^6
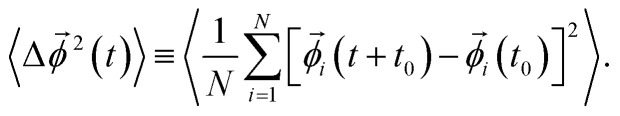


**Fig. 9:**
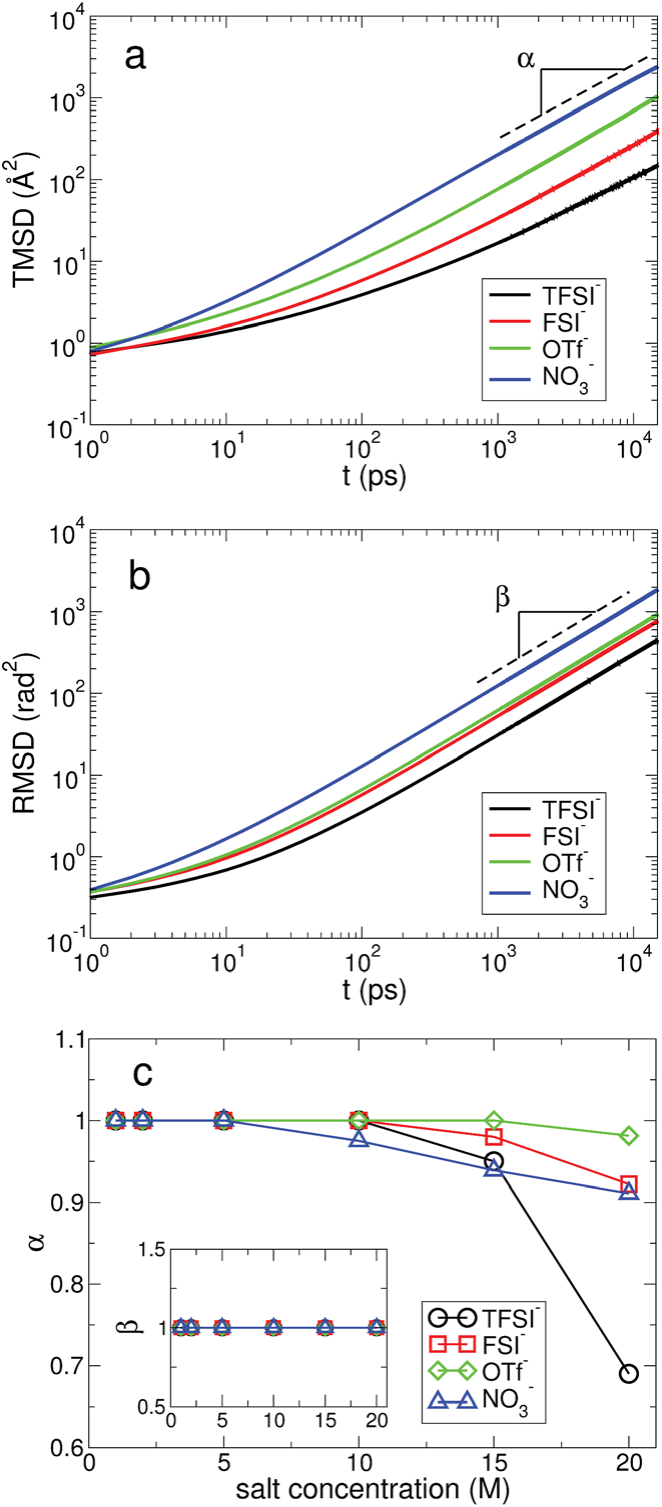
Mean square displacement of water molecules. (a) The translational mean square displacement (TMSD) of water molecules at the salt concentration of 20 M for different anions. (b) The rotational mean square displacement (RMSD) of water molecules at the salt concentration of 20 M for different anions. (c) The exponent *α* of time *t* in the TMSD (∝*t*^*α*^) in the long time limit as a function of salt concentration for different anions. Inset: the exponent *β* of time *t* in the RMSD (∝*t*^*β*^) in the long time limit as a function of salt concentration for different anions.

In Fig. 9, we present the TMSD and RMSD of water molecules in the superconcentrated limit in the presence of the different types of anion. For bulk water, the dynamic characteristics of water exhibit diffusive motion for both the translational and rotational dynamics in the long time limit,^57,58^ showing both exponents *α* = *β* = 1 in the relations 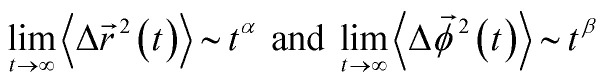. In the superconcentrated limit, we find that *α* is less than 1, indicating the possibility of subdiffusive motion (*α* < 1) of water molecules. The subdiffusive translational motion of water has been observed in single-file diffusion inside a narrow carbon nanotube^59–61^ and it represents strongly correlated dynamics. In contrast, we find that *β* is always equal to 1 for all salt concentrations and for all anion species we investigated, showing that the rotational motion of water molecules remains diffusive, the same as in bulk water. This indicates that in superconcentrated aqueous electrolytes the temporal behavior of the translational and rotational motions could be separated (or decoupled). For the translational motion, the magnitude of *α* shows TFSI^−^ < NO_3_^−^ < FSI^−^ < OTf^−^. For the three anions except TFSI^−^, the value of *α* is placed at between 0.9 and 1.0. As a result, we can interpret that it might represent simply a small deviation from the diffusive motion or weak subdiffusive motion. For TFSI^−^, the translational motion of water clearly shows subdiffusive motion with *α* = 0.69 in the superconcentrated limit.^24^ Thus, we conclude that the occurrence of subdiffusive translational motion could depend on the type of anion.

Using the results of the TMSD and RMSD, we examine the behavior of the translational diffusion constant *D*_T_ and the rotational diffusion constant *D*_R_ of water molecules for the different types of anion. We calculate *D*_T_ from the TMSD *via* the Einstein relation,^24,43,44,51,54^7
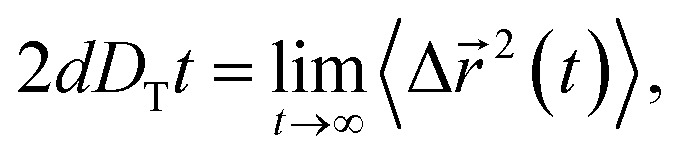
where *d* is the dimensionality of the system. For the translational motion, the *D*_T_ of water molecules for all anions shows an exponential decrease upon raising the salt concentration. As the salt concentration increases, the ion–water interaction will increase, so that it causes an increase in the rigidity of the hydration shell. It finally entails an increase in the drag to the motion of water molecules similar to the dynamic response to pressure.^62^ Interestingly, we find a crossing behavior in the dependence of *D*_T_ on the salt concentration with respect to the anion type. Up to 5 M, *D*_T_ is the largest with FSI^−^, but above 5 M *D*_T_ becomes the largest with NO_3_^−^. We also find similar crossing behaviors in the other dynamical properties such as the slow HB dynamics with anions. In the anionic environment with FSI^−^, the rigidity of the anionic hydration shell is weakest among the anions on both the short and long timescales. As shown in Fig. 7, the rigidity of the cationic hydration shell with FSI^−^ is weaker than those of TFSI^−^ and OTf^−^, but similar to that of NO_3_^−^. And it rapidly increases for higher salt concentrations. Hence the translational motion of water molecules with FSI^−^ rapidly slows down and the crossing could occur at a certain concentration.

In a similar way, we calculate *D*_R_ from the RMSD,^24,56,57,63^8
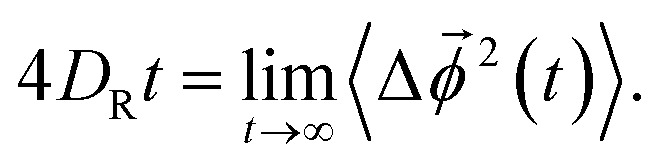


**Fig. 10:**
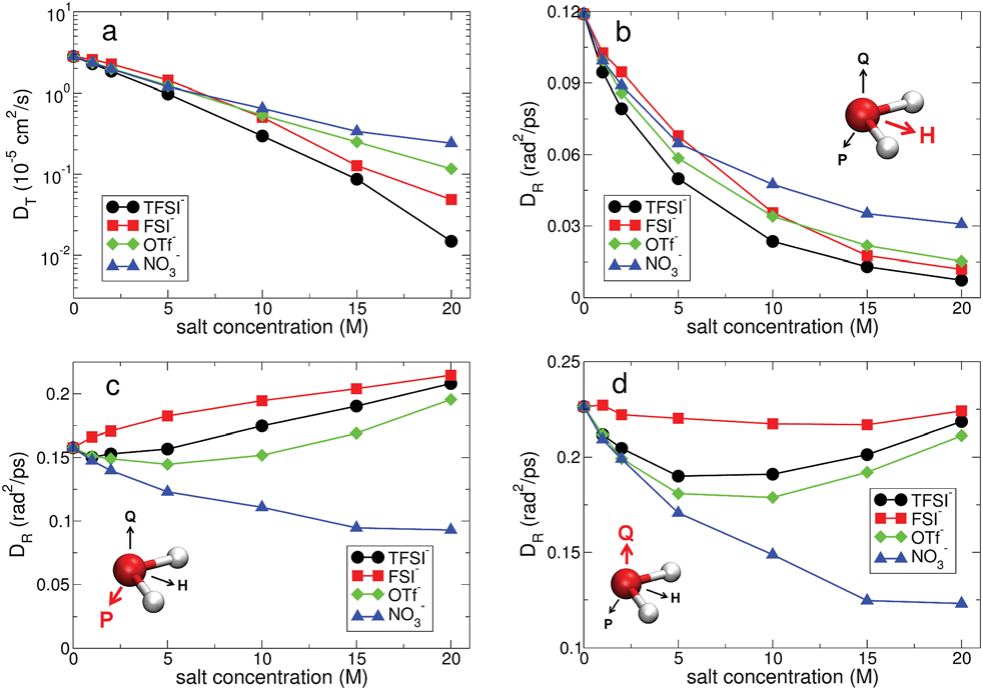
Translational and rotational diffusion constants of water molecules. (a) Translational diffusion constant *D*_T_ of water molecules as a function of salt concentration for different anions in a semi-log plot. For subdiffusive translations, we estimate *D*_T_ by simply calculating the slope of TMSD (divided by 6) in Fig. 9. (b) Rotational diffusion constant *D*_R,H_ of a polarization vector *H⃑* of a water molecule as a function of salt concentration for different anions. (c) Rotational diffusion constant *D*_R,P_ of a principal vector *P⃑* of a water molecule as a function of salt concentration. (d) Rotational diffusion constant *D*_R,Q_ of a principal vector *Q⃑* of a water molecule as a function of salt concentration.

To fully quantify the rotational dynamics of water molecules, we additionally calculate the rotational diffusion constants of the other two normalized principal vectors, *P⃑*(*t*) and *Q⃑*(*t*),^24,56,57^ as shown in Fig. 10. For *D*_R,H_, the rotational motion of water molecules decreases upon increasing the salt concentration, representing the stronger attractive interaction between a Li^+^ ion and the oxygen atom of water molecules for higher salt concentrations. For *D*_R,P_, we can categorize the rotational motion of water molecules into two groups according to the type of anions. For TFSI^−^, FSI^−^ and OTf^−^, *D*_R,P_ in the superconcentrated limit is larger than *D*_R,P_ at low concentration. This behavior of *D*_R,P_ is in contrast to *D*_R,H_ with respect to the salt concentration, indicating an occurrence of anisotropy in the rotational motion of water molecules.^24^ Namely, water molecules rotate more slowly in a certain molecular direction but rotate faster in the other molecular directions. Anisotropy in the rotational motion of water has been also found in water in polymer networks and around ions,^64,65^ emphasizing the effect of the complex environment. The rotational motions of a water molecule in all three directions cannot be independent, so that *D*_R,Q_ shows the combined behaviors of *D*_R,H_ and *D*_R,P_. Interestingly, we do not find the anisotropic rotation of water molecules with NO_3_^−^. For all three rotations *D*_R_ decreases upon increasing the salt concentration. Thus, we conclude that the occurrence of the anisotropic rotation of water molecules depends on the type of anion. For NO_3_^−^, water molecules in the superconcentrated limit can still be hydrogen-bonded to each other by simultaneously acting as one HB donor and one HB acceptor, even though a large amount of ions exists. This affects how water molecules rotate in the surrounding conditions with NO_3_^−^.

## 4 Conclusions

When ions are dissolved in water, the structural and dynamic properties of water deviate from neat water depending on the ion–water interaction. For bulk water, the complex HB network structure spans the whole space and it critically determines the unique and unusual characteristics of water. As the amount of ions increases, the ion–water interaction becomes significant instead of the water–water interaction. The HB network in water would be interrupted by ions and the disrupted HB network would be also different from bulk water. For a dilute concentration, the presence of ions can be treated as a perturbation to the properties of bulk water. In the superconcentrated limit, however, the situation surrounding the water molecules cannot be simply described by the perturbative ion–water interaction. In this case, the existence of the full HB connectivity in water is generally obscured. Since the structure of the system will be mainly affected by ions, the simple role of ions as structure-makers or structure-breakers in the HB network has to be modified. The local electric field induced by cations and anions will suppress or enhance the translational and rotational dynamics of water molecules. Because the ion–water interaction also depends on the type of ions, the ion type would be of great importance to the properties of water.

In this work, we have performed molecular dynamics simulations to investigate the effects of anions on the structural and dynamical properties of water for various salt concentrations, especially focusing on superconcentrated conditions. For most structural properties of water, we found that the anionic effects at low concentration are negligible. In other words, the existence of different anions does not affect the structural properties of water at low concentration. We found that at the low salt concentration of 1 M the HB features in water, such as the HB structure and HB dynamics, do not exhibit any dependence on the anion species as well. The different properties of water in the presence of different anions appear above a certain concentration of ions. In the superconcentrated limit, most properties of water show a strong dependence on the type of anion. For the anionic hydration dynamics as well as the HB dynamics of water, we found that there are two opposite behaviors with respect to the salt concentration and two timescales are needed for fully describing the dependence of the fast and slow dynamics on the salt concentration. In contrast, for the hydration dynamics associated with an Li cation we observed the same behavior of the cationic hydration dynamics on short and long timescales. Those different behaviors are ascribed to the different mechanisms to form and break the bonds of water molecules with cations and anions. In addition, the existence of different anions would affect the translational and rotational dynamics of water molecules, such as the appearance of the subdiffusive translation and anisotropic rotation of water molecules.

We believe that our results will provide a comprehensive molecular picture on the effects of anions on aqueous electrolytes for a wide range of salt concentrations. Particularly, our results will give broader understanding of the properties of water in various environments. Even though our results are based on the lithium cation, we think that our results are able to be extended to more general cases. Finally, we believe that our results will give an insight into chemical and biological processes as well as lithium ion batteries.

## Conflicts of interest

There are no conflicts to declare.

## Supplementary Material
